# Safety and feasibility of dual antiplatelet therapy termination guided by angioscopic findings 6 months after flow diverter placement

**DOI:** 10.3389/fneur.2025.1649290

**Published:** 2025-08-29

**Authors:** Kenji Fukutome, Shuta Aketa, Junji Fukumori, Taigi Fujita, Motoki Fukunaga, Yuki Shiraishi, Atsuko Shimotsuma, Yasutaka Murakami, Ryuta Matsuoka, Mikio Shiba, Rinsei Tei, Yasushi Shin, Yoshiharu Higuchi, Yasushi Motoyama

**Affiliations:** ^1^Department of Neurosurgery, Osaka Keisatsu Hospital, Osaka, Japan; ^2^Department of Neurology, Osaka Keisatsu Hospital, Osaka, Japan; ^3^Cardiovascular Division, Osaka Keisatsu Hospital, Osaka, Japan

**Keywords:** angiography, angioscopy, aneurysm, cone-beam computed tomography, dual antiplatelet therapy, flow diverter, neointima

## Abstract

**Background:**

Flow diverters (FDs) are commonly used to treat intracranial aneurysms with wide necks. Dual antiplatelet therapy (DAPT) is essential during the perioperative period to prevent thrombosis; however, it increases the risk of hemorrhagic complications, warranting early discontinuation when feasible. Neointimal formation over FD is crucial for safe DAPT discontinuation. This study aimed to directly visualize neointimal coverage 6 months after FD placement using angioscopy and evaluate the safety of DAPT termination.

**Methods:**

Eight consecutive patients undergoing FD placement for internal carotid artery aneurysms between April 2022 and February 2024 were included in the prospective evaluation. Angioscopy was conducted at 6 months (±1 month) after FD placement to assess neointimal formation, which was graded 0–3 based on coverage (grade 0: no neointima; grade 1: slight; grade 2: translucent coverage; grade 3: full opaque coverage). Cone-beam computed tomography (CT) was concurrently performed to evaluate radiolucent gaps as indirect evidence of neointima formation. DAPT was discontinued if the neointimal coverage was graded ≥1, followed by the monitoring of ischemic events for 1 month.

**Results:**

The mean age of patients was 60.5 (49–81) years, and the mean aneurysm diameter was 7.7 mm (5.1–14.6 mm). Angioscopic neointimal grading was ≥1 in all cases, while cone-beam CT revealed no radiolucent gaps in one case. No procedural complications were observed. Following DAPT discontinuation, all the patients were administered single antiplatelet therapy, with no ischemic events observed within 1 month.

**Conclusion:**

Angioscopy reliably confirmed neointimal coverage 6 months after FD placement, suggesting the potential for safe DAPT discontinuation. The findings underscore the superiority of angioscopy over cone-beam CT in identifying thin neointima. Further studies involving larger cohorts and applying advanced imaging technologies are required to optimize post-FD antiplatelet therapy.

## 1 Introduction

Flow diverter (FD) is commonly used to treat intracranial aneurysms with a wide neck. During the perioperative period of FD placement, dual antiplatelet therapy (DAPT) is administered to prevent thrombosis (1, 2). However, DAPT increases the risk of hemorrhagic complications, which indicate an early DAPT discontinuation (3). No standard scheme is available for antiplatelet therapy after FD placement, including the combination and duration of the medication therapy, in which the reported practices vary by facility (4).

DAPT can be discontinued, if a stent lumen is sufficiently covered by neointima. Neointimal coverage after FD placement can be evaluated with cone-beam computed tomography (CT) (5); however, its detection is challenging when the neointima is very thin. In this study, we used angioscopy to evaluate lesions under a direct view, which allowed us to visualize thin neointima after carotid artery stenting or FD placement (6–8).

We previously reported a case where adequate neointima formation was detected in the angioscopy performed 6 months after FD placement, allowing for safe DAPT discontinuation (7). However, comprehensive studies on the gross evaluation of neointimal formation following FD placement are lacking. This study aimed to confirm neointimal formation 6 months after FD placement using angioscopy, followed by DAPT completion, to predict ischemic events. The findings were compared with those obtained using cone-beam CT.

## 2 Materials and methods

The authors declare that all the supporting data is available in the article.

### 2.1 Patient selection

This observational study conducted at Osaka Keisatsu Hospital between April 2022 and February 2024 included eight consecutive patients who underwent FD placement for internal carotid artery (ICA) aneurysms. The proximal end of FD was positioned proximal to the C4/5 segment. The entire FD was fully adjusted to the arterial wall, using a balloon if necessary.

### 2.2 Follow-up protocol

Neointimal formation was evaluated by angioscopy 6 months (± 1 month) after FD placement. Concurrently, biplane angiography was used to detect radiolucent gaps between the stent and vessel lumen, which indicate neointimal formation (6). DAPT was administered at least 2 weeks before FD placement and continued until angioscopic examination. As antiplatelet agents, aspirin (100 mg/day) and prasugrel (3.75 mg/day) were included in the therapeutic regimen. If neointima was detected by angioscopy (grading score ≥ 1), DAPT was ceased, and the patient was followed up for ischemic events within 1 month. Aspirin was used as a single agent for the reduction to single antiplatelet therapy (SAPT) but was replaced by prasugrel when ineffective, as determined by functional testing. The antiplatelet functional tests were performed using Hematracer ZEN (DS Medical, Tokyo, Japan), with a class of ≤4 indicating sufficient drug efficacy.

### 2.3 Angioscopic procedures

An 8-Fr sheath was inserted into a femoral artery under local anesthesia, followed by the intravenous administration of 3,000 units of heparin. The 8-Fr balloon catheter was placed in the ICA on the FD side using a guidewire and inner catheter. First, biplane angiography was performed to evaluate the aneurysm status using the O’Kelly–Marrota (OKM) Grading Scale (9). The cone-beam CT with 20% diluted contrast was performed to confirm the presence of a radiolucent gap, which indirectly indicated neointimal formation (6). Cone-beam CT (CBCT) images were acquired using an interventional angiography unit with a 30 × 30 cm detector (Innova™ IGS630, GE Healthcare, Chicago, IL). In our CBCT acquisition protocol, at 12 × 12 cm FOV, a 50 fps × 13 s scan with 200° rotations of the flat-panel detector around a patient was obtained. Diluted contrast was injected at 3 mL/s for a total 39 mL (+ *α* mL based on the proper X-ray delay time owing to the patient’s condition). 3D reconstructions were conducted on a workstation V_S 7, generating a 5,123-voxel matrix. Subsequently, a 5-Fr Navien distal access catheter (Medtronic, Minneapolis, MN) was advanced to the FD’s proximal portion, using a microguidewire and Phenom 27 microcatheter (Medtronic). The VISIBLE angioscope (INTER-TEC MEDICALS, Osaka, Japan) was then approximated to the Navien tip. With a balloon blocking the proximal blood flow, heparinized saline was manually infused from two catheters to clear arterial blood, allowing the direct visualization of neointima (10). Based on previous findings, neointima was graded from 0 to 3 by two neurosurgeons (KF and SA). The assessments of both observers were in full agreement, with no discrepancies noted. Grade 0 stands for a visible stent strut without any neointima visualized; grade 1 reflects slight neointimal formation with visible stent struts; grade 2 represents fully covered stent struts with translucent visualization of their structure; and grade 3 displays full coverage of a stent strut without visible configuration (11). Following the observation of the flow diverter along the maximum feasible extent–albeit limited in actual coverage–the procedure was concluded by removing the sheath with a hemostatic device.

### 2.4 Ethics statement

The procedures performed were approved by the ethics committee of the Osaka Keisatsu Hospital, and patients provided their informed consent.

## 3 Results

**Figure 1:**
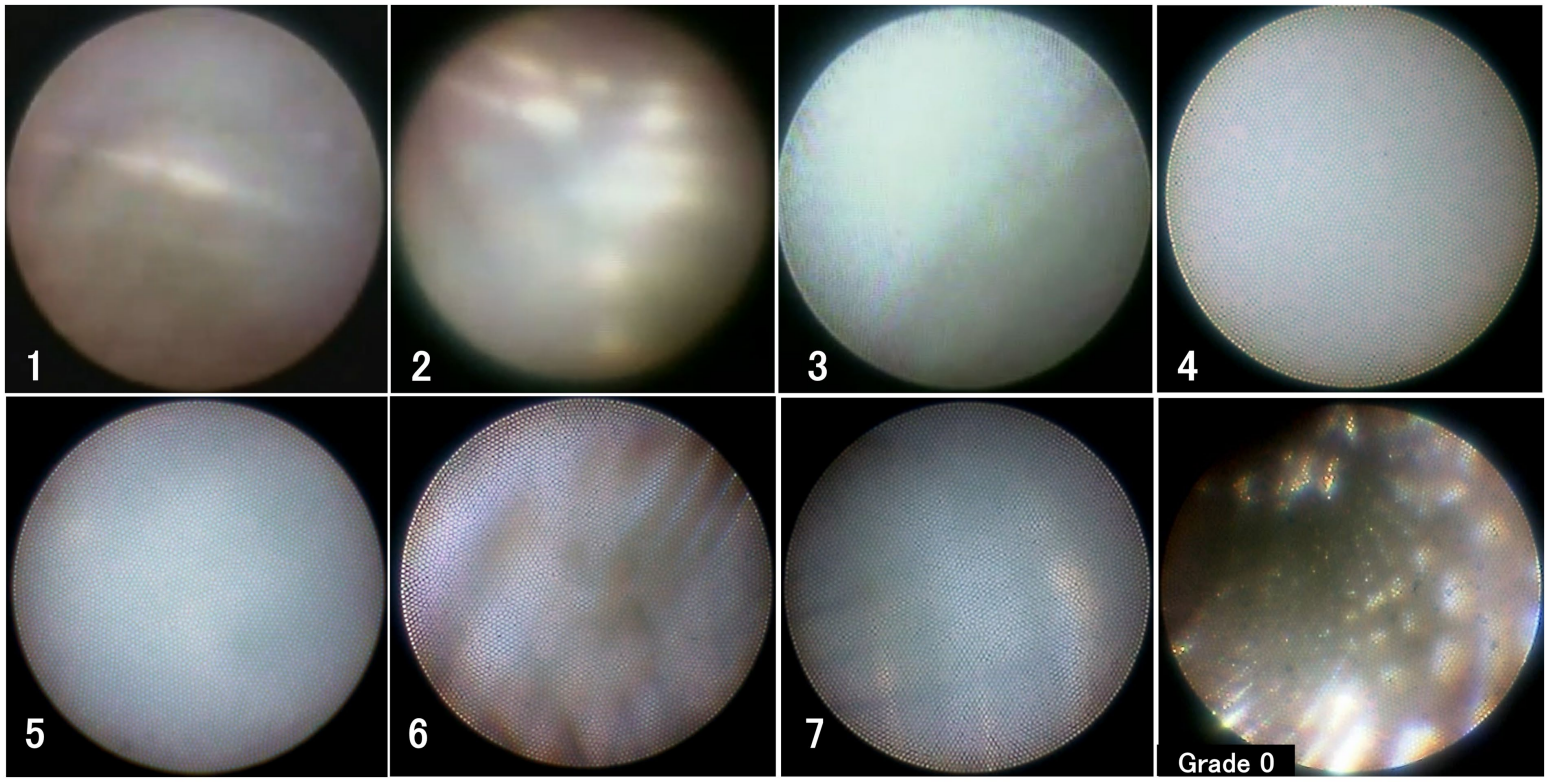
Angioscopic images. The numbers indicate the respective case numbers. The lower right shows an angioscopic image of a sample PIPELINE Flex as a reference for grade 0.

The results are summarized in Table 1. The mean age of the eight participants was 60.5 (49–81) years, and three of them were male. The mean maximum diameter of aneurysms was 7.7 mm (5.1—14.6 mm), and PIPELINE Flex with Shield Technology (Medtronic) was used for FD placement in all cases. The radiolucent gap on cone-beam CT was incomplete or absent in all but one case, while the neointimal coverage graded by angioscopy was 1 or higher in all cases (Figure 1, except case 8). Complications associated with the series of procedures were not detected in any patients. Aspirin therapy was effective in all patients, as confirmed by antiplatelet function tests, and all the regimens were reduced to aspirin alone, with no ipsilateral ischemic complications observed within 1 month.

**Table 1:** Patient characteristics and outcomes.

	Age (yr)/Gender	Side	Maximum diameter (mm)	DAPT	Flow diverter	Neointimal coverage grading	Radiolucent gap	OKM grading	SAPT	Ischemic events
1	61/Man	Left	14.6	ASA + PSG	PIPELINE Flex with ST	1	Incomplete	C	ASA	No
2	69/Woman	Right	5.7	ASA + PSG	PIPELINE Flex with ST	1	Incomplete	B	ASA	No
3	52/Man	Right	5.2	ASA + PSG	PIPELINE Flex with ST	2	Complete	D	ASA	No
4	51/Woman	Right	6.4	ASA + PSG	PIPELINE Flex with ST	3	Incomplete	D	ASA	No
5	60/Woman	Right	5.1	ASA + PSG	PIPELINE Flex with ST	2	Incomplete	D	ASA	No
6	49/Man	Left	5.9	ASA + PSG	PIPELINE Flex with ST	1	Incomplete	B	ASA	No
7	61/Woman	Right	5.1	ASA + PSG	PIPELINE Flex with ST	1	Incomplete	D	ASA	No
8	81/Woman	Right	13.8	ASA + PSG	PIPELINE Flex with ST	1	None	C	ASA	No

ASA, acetylsalicylic acid; DAPT, dual antiplatelet therapy; OKM, O’Kelly-Matotta; PSG, prasugrel; SAPT, single antiplatelet therapy; ST, Shield Technology.

### 3.1 Representative case (case 8)

**Figure 2:**
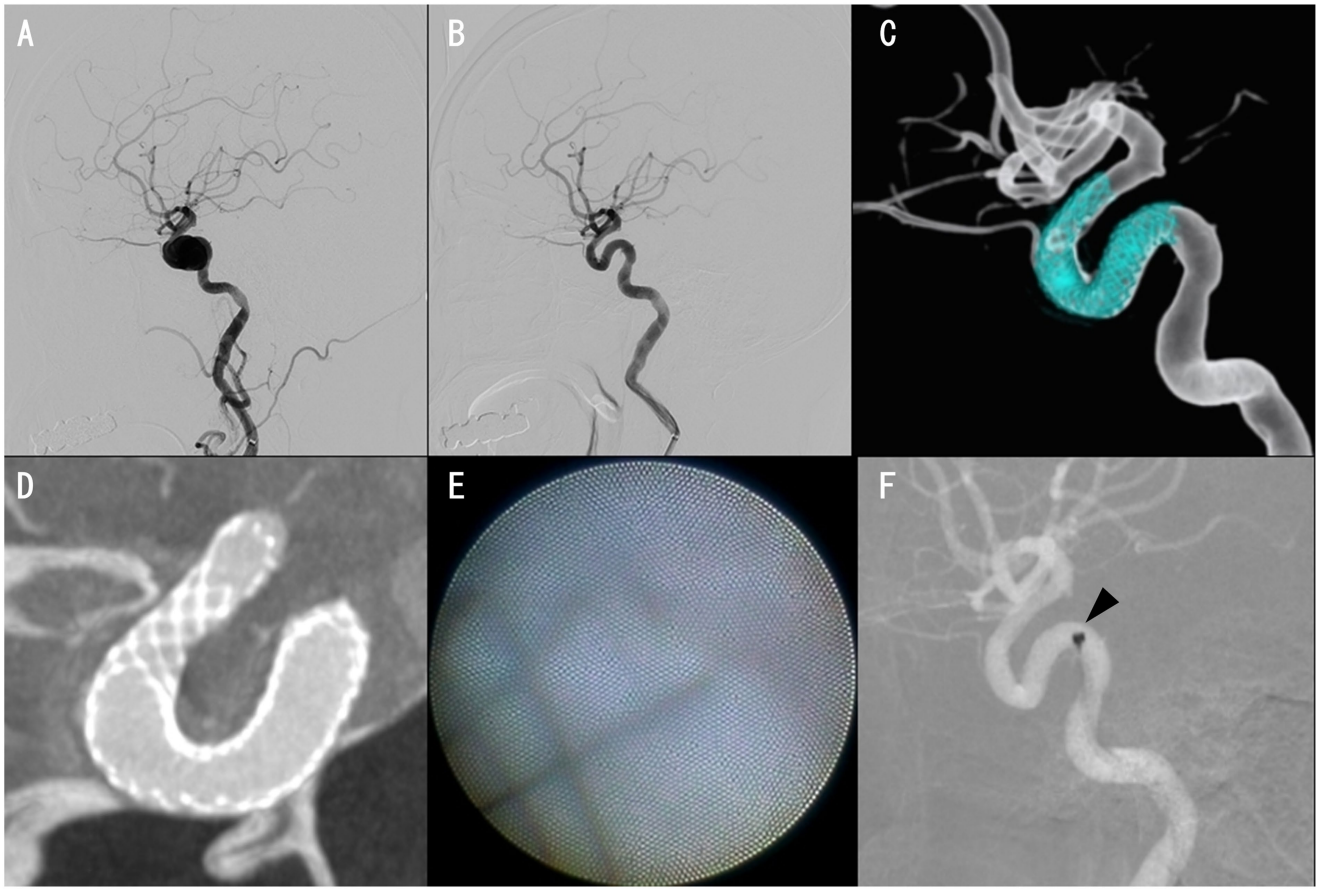
Case 8 images. Preinterventional right internal carotid angiography (ICAG) displaying cavernous ICA aneurysm **(A)**. The aneurysm was almost occluded, with only a small amount of blood flow near the neck in the right ICAG 6 months after placing a PIPELINE Flex with shield technology **(B,C)**. Cone-beam CT with 20% diluted contrast exhibiting no apparent radiolucent gap **(D)**. Proximal edge of the PIPELINE Flex covered with thin neointima **(E)**. Angioscope position, where the image reflected in E was obtained (**F**, arrowhead).

Case 8 was an 81-year-old woman. The PIPELINE Flex with Shield Technology was placed, followed by full-length percutaneous transluminal angioplasty for an asymptomatic unruptured cavernous ICA aneurysm with a maximum diameter of 13.8 mm (Figures 2A–C). Cone-beam CT with 20% diluted contrast did not display any radiolucent gap (Figure 2D), but the angioscopy revealed grade 1 neointima (Figures 2E, F). Based on these results, DAPT was terminated, and the regimen was continued on aspirin alone, with no ischemic events experienced by the patient over the following month.

## 4 Discussion

The timing of neointimal formation after FD placement is poorly understood. The growth of immature amorphous endothelial cells has been reported in canine coronary arteries 1 week after stenting (12). For FD, optical coherence tomography (OCT) confirmed neointimal development as early as 7 days after PIPELINE placement, albeit in a porcine model (13). For living humans, Guerrero et al. confirmed neointimal formation by OCT 8 weeks after FD placement (14). Other methods to assess neointimal formation have been demonstrated, including cone-beam CT and angiography (15, 16); however, all visualizations were indirect evidence of neointima.

All the cases in this study involved PIPELINE Flex with Shield Technology, and neointima formation could be directly visualized by angioscopy 6 months after FD placement for all patients. These results confirmed that sufficient neointimal formation (grade ≥ 1) occurred 6 months after FD placement. Based on the abovementioned report, this formation may have happened even earlier. Angioscopy is considered to have superior properties for identifying thin neointima. OCT is characterized by an excellent resolution, but it requires a lesion cross, making it difficult to implement in a siphon section of an ICA with strong tortuousness. Because both examinations are highly invasive, this study compared them with cone-beam CT using diluted contrast for future application; however, CT did not allow the detection of very thin neointima (Case 8). A case report of intimal hyperplasia after FD placement was previously observed using magnetic resonance vessel wall imaging (17), but thin neointima identification is thought to be equally difficult with that method. The detection of ultra-thin neointima awaits the advent of higher resolution angiography, CT, and magnetic resonance imaging.

DAPT duration after FD placement has been actively discussed (3, 18, 19). It is easy to assume that DAPT can be ceased if sufficient neointima is covering an entire stent, including FD. We previously reported a case where sufficient neointimal coverage was confirmed by angioscopy 2 months after CAS, and DAPT was terminated without further complications (8). However, no studies, including systemic ones, have actually verified whether neointimal coverage after stent placement can be directly visualized, allowing a safe DAPT termination. This study has also demonstrated that DAPT could be safely terminated when adequate neointimal coverage occurred.

In recent years, FDs with anti-thrombogenic surface modifications, such as PIPELINE Flex with Shield Technology using 2-methacryloyloxyethyl phosphorylcholine polymer, have been distributed, and discontinuing DAPT regardless of neointimal formation may be possible (20, 21). Furthermore, SAPT is sufficient from the start of antithrombotic therapy for FDs with anti-thrombogenic surface modifications (22, 23), eliminating further need for DAPT.

Our study exclusively utilized the PIPELINE Flex with Shield Technology, a coated flow diverter known for its anti-thrombogenic surface modification. The adequate neointimalization observed at 6 months after using this device aligns with previous findings suggesting faster endothelialization with the use of surface-modified FDs. However, it remains unclear whether uncoated FDs require a longer period for complete neointimal maturation, potentially necessitating an extended DAPT duration. Preclinical and clinical evidence suggests that uncoated devices may exhibit different healing characteristics, which could impact the optimal antiplatelet strategy. Therefore, comprehensive comparative studies assessing the neointimal coverage across various FD types (coated vs. uncoated) at different time points are crucial to provide evidence-based recommendations for tailored DAPT regimens.

Although our study demonstrates the utility of angioscopy in assessing neointimal coverage at 6 months post-FD placement and suggests the feasibility of DAPT termination based on these findings, it does not conclusively establish 6 months as the optimal duration of DAPT. Determining the ideal DAPT duration would necessitate a comparative assessment of neointimal healing across varying DAPT timeframes, which was beyond the scope of this preliminary study. Therefore, further research involving such comparative studies is crucial to definitively ascertain the optimal DAPT regimen.

This study has some limitations. First, the recruited cohort is relatively small. Although a larger number of cases of internal carotid artery aneurysms treated with FD might generally be available, our study specifically included only cases where the proximal end of the FD was positioned proximal to the C4/5 segment. This selection criterion was adopted because the angioscope could not be advanced beyond this segment due to its rigidity and the internal carotid artery’s tortuousness, thereby inherently limiting the number of eligible patients for a consistent angioscopic evaluation. Therefore, although our study findings suggest that DAPT could potentially be safely terminated when adequate neointimal coverage is observed via angioscopy, this conclusion is based on a limited sample size of eight patients and requires further validation in larger, more comprehensive studies. The generalizability of these findings is thus restricted. Moreover, the single-center nature of the present study, coupled with the small sample size and exclusive use of Pipeline Flex with Shield Technology, raises concerns about a potential selection or publication bias. Second, the type of FDs used was not uniform, but coincidentally, the only FDs used were Pipeline Flex with Shield Technology, so the results obtained in this study do not apply to FDs in general. Third, VISIBLE angioscope is one of the slimmest available in clinical practice, but it is rigid and cannot go beyond a siphon of ICA; therefore, neointima was identified only at the proximal end of the FD. Although a uniform neointimal coverage across the entire device cannot be conclusively demonstrated, its presence may be inferred based on the observed findings. A very slim microangioscope has been developed at the preclinical stage, which, if used clinically, can allow the evaluation of neointima throughout the FD and may aid in antiplatelet drug management (24, 25). Fourth, the follow-up at 1 month after DAPT termination was done only for clinical symptoms, while no imaging studies were performed. Our study aimed to assess the immediate safety of transitioning from DAPT to SAPT when angioscopic neointimal coverage was confirmed. All enrolled patients received effective aspirin therapy as confirmed by the functional test results. However, the 1-month follow-up duration for ischemic events post-DAPT discontinuation appears insufficient, as delayed complications, including in-stent stenosis, may occur beyond this window. The primary focus of the present study was on the immediate post-DAPT discontinuation period, and the limited follow-up duration reflects this scope. Therefore, long-term monitoring is critical, and future studies should incorporate extended follow-up durations to capture such delayed events and provide a more comprehensive safety assessment. Fifth, although angioscopy offers a direct visualization of neointimal coverage, the present study did not assess its diagnostic performance metrics, including sensitivity and specificity. Such evaluation typically requires comparison with a gold standard (e.g., histological analysis), which was not feasible in our clinical setting. Future studies should aim to establish these metrics to further validate the diagnostic reliability of angioscopy for neointimal assessment. To address these limitations, future large-scale, multi-center studies involving various types of flow diverters are essential to further optimize post-FD antiplatelet therapy and mitigate the risk of such biases.

In conclusion, this study demonstrates that neointimal coverage can be reliably confirmed using angioscopy 6 months after PIPELINE Flex with Shield Technology placement. Our findings tentatively suggest that DAPT may be safely terminated when sufficient neointimal formation is directly visualized. The comparison with cone-beam CT highlights the superiority of angioscopy in detecting thin neointima and providing a potential pathway for tailored antiplatelet regimens. However, the limitations, such as the small sample size, the exclusive use of Pipeline Flex with Shield Technology, and the inability to evaluate the entire stent, underscore the need for further studies. Future advancements in angioscopic and imaging technologies may enable the comprehensive evaluation of neointimal coverage and further refine post-FD antithrombotic therapy strategies.

## Data Availability

The raw data supporting the conclusions of this article will be made available by the authors, without undue reservation.
